# Supplementary material for: Strategic coastal dike shape for enhanced tsunami overflow reduction
Source: PLoS One. 2023 Oct 13;18(10):e0292930. doi: 10.1371/journal.pone.0292930 (PMC10575497; doi:10.1371/journal.pone.0292930)
Supplement: Attachment — Submitted filename: Response to Reviewers.docx [file pone.0292930.s002.docx]

**PLOS ONE**

**Manuscript Number**: PONE-D-23-18971

**Authors**: TAKEGAWA Naoki, SAWADA Yutaka, FURUICHI Noriyuki

**Title**: Strategic Coastal Dike Shape for Enhanced Tsunami Overflow Reduction

Reviewer 1

|  | We wish to express our appreciation to the reviewer for his or her insightful comments, which have helped us significantly improve the paper. |
| --- | --- |
| 1-1) | Comment  Abstract should incorporate the problems under investigation, purpose, methodology used, finding and recommendations. However, this paper failed to include some major components. Therefore, missed components should be included. |
|  | Response  As you pointed out, the abstract was missing the presentation of the problem, the purpose, and the recommendations. We have now added these details to the abstract (L.11-13, L.19-20). |
| 1-2) | Comment  Key words should be included after the abstract in the main manuscript. |
|  | Response  We have added key words after the abstract in the main manuscript (L.23-24). |
| 1-3) | Comment  The discussion part of the paper is shallow. |
|  | Response  We have divided the discussion section into three parts and expanded on each, enriching the content (L.273-331). |
| 1-4) | Comment  Since the study will have paramount significance recommendation should be included after the conclusion. |
|  | Response  We appreciate your suggestion to include recommendations after the conclusion, emphasizing the paramount significance of the study. In response to your comment, we have restructured the "Conclusions" section to clearly differentiate between our "Key Findings" and "Recommendations". We believe that this new structure provides a clearer distinction and emphasizes the importance of our "Recommendations", as you rightly pointed out. (L.344-358) |

Reviewer 2

|  | We wish to express our appreciation to the reviewer for his or her insightful comments, which have helped us significantly improve the paper. |
| --- | --- |
| 2-1) | Comment  It is better to start the first part of the summary of the article with the problem statement and then state the objectives of the current study in order to solve the aforementioned problems. |
|  | Response  We agree with the feedback provided. We have started the abstract with the current situation and the posed problem (L.11-13). |
| 2-2) | Comment  Please consider rewriting keywords according to National Library of Medicine terms accessible at: <https://meshb.nlm.nih.gov> |
|  | Response  We have reconsider key words according to the NLM terms accessible. Only the keyword "coastal dike" is not registered in the NLM. We have included "coastal dike" because it is an important keyword for this study (L.22-23). |
| 2-3) | Comment  It is reasonable to describe numerical simulations as a scientific technique before stating it's use in this study at the first paragraph. |
|  | Response  The use of numerical simulation in this study has been added in each relevant section (L.56, L.67-68). |
| 2-4) | Comment  Please state the limitations of the current study and possible limitations or errors in using the desired technique. (If there is no limit, it should be mentioned.) |
|  | Response  The points to be cautious about (limitations or errors) are the “effectiveness for tsunamis with longer periods” and the “durability and stability of the dike itself”. We have elaborated on these points in more detail in the Discussion section (L.274-300, L.316-331). |

Reviewer 3

|  | We wish to express our appreciation to the reviewer for his or her insightful comments, which have helped us significantly improve the paper. |
| --- | --- |
| 3-1) | Comment  There were no analysis statistical method used in the study. It should be explained clear in the Method. |
|  | Response  Thank you for pointing out the absence of statistical methods in our Methods section. The core reason for this is that our results were derived without the use of intricate statistical method. Our numerical analysis is based on a deterministic model. In this study, we achieved our objective of verifying the dike shape against the assumed conditions without resorting to statistical methods (L.119-157). Specifically, the results from our simulations provide water levels, flow velocities, and pressures at various times within the simulation domain. We then directly used these outputs to create graphs illustrating the flow fields around the coastal dike and the amount of the overflow. However, we recognize that in future developments, when constructing design methodologies, there might be a need to incorporate statistical analysis, especially as tsunami conditions can vary probabilistically. To enhance clarity, the Methods section of the revised manuscript now includes a more detailed description of the simulation and its future implications (L.92-98). |
| 3-2) | Comment  All another studies had been explained clearly in the study. |
|  | Response  Thank you for acknowledging the clarity with which we explained other studies in our manuscript. We aimed to provide comprehensive and clear descriptions to assist readers in understanding the context and relevance of our work. |
| 3-3) | Comment  The research should put the weakness of using dike shape. |
|  | Response  In response to the reviewer's comment, when utilizing the findings from our study, the points of caution (weaknesses) in our study are the “effectiveness for tsunamis with longer periods” and the “durability and stability of the dike itself”. We have elaborated on these points in more detail in the Discussion section (L.274-300, L.316-331). |
